# LRP1 Has a Predominant Role in Production over Clearance of Aβ in a Mouse Model of Alzheimer’s Disease

**DOI:** 10.1007/s12035-019-1594-2

**Published:** 2019-04-19

**Authors:** Bart Van Gool, Steffen E. Storck, Sara M. Reekmans, Benoit Lechat, Philip L. S. M. Gordts, Laurent Pradier, Claus U. Pietrzik, Anton J. M. Roebroek

**Affiliations:** 1 Laboratory for Experimental Mouse Genetics, Department of Human Genetics, KU Leuven, Herestraat 49, Box 604, 3000 Leuven, Belgium; 2 Institute for Pathobiochemistry, University Medical Center, Johannes Gutenberg University of Mainz, Mainz, Germany; 3 Department of Medicine, Division of Endocrinology and Metabolism, University of California San Diego, La Jolla, CA 92093 USA; 4 SANOFI, Neuroscience Therapeutic Area, 1 Avenue P. Brossolette, 91385 Chilly-Mazarin, France

**Keywords:** Alzheimer’s disease, Animal model, APP, LRP1, APP metabolism, Aβ clearance

## Abstract

The low-density lipoprotein receptor-related protein-1 (LRP1) has a dual role in the metabolism of the amyloid precursor protein (APP). In cellular models, LRP1 enhances amyloid-β (Aβ) generation via APP internalization and thus its amyloidogenic processing. However, conditional knock-out studies in mice define LRP1 as an important mediator for the clearance of extracellular Aβ from brain via cellular degradation or transcytosis across the blood-brain barrier (BBB). In order to analyze the net effect of LRP1 on production and clearance of Aβ in vivo, we crossed mice with impaired LRP1 function with a mouse model of Alzheimer’s disease (AD). Analysis of Aβ metabolism showed that, despite reduced Aβ clearance due to LRP1 inactivation in vivo, less Aβ was found in cerebrospinal fluid (CSF) and brain interstitial fluid (ISF). Further analysis of APP metabolism revealed that impairment of LRP1 in vivo shifted APP processing from the Aβ-generating amyloidogenic cleavage by beta-secretase to the non-amyloidogenic processing by alpha-secretase as shown by a decrease in extracellular Aβ and an increase of soluble APP-α (sAPP-α). This shift in APP processing resulted in overall lower Aβ levels and a reduction in plaque burden. Here, we present for the first time clear in vivo evidence that global impairment of LRP1’s endocytosis function favors non-amyloidogenic processing of APP due to its reduced internalization and subsequently, reduced amyloidogenic processing. By inactivation of LRP1, the inhibitory effect on Aβ generation overrules the simultaneous impaired Aβ clearance, resulting in less extracellular Aβ and reduced plaque deposition in a mouse model of AD.

**Electronic supplementary material:**

The online version of this article (10.1007/s12035-019-1594-2) contains supplementary material, which is available to authorized users.

## Introduction

One of the hallmarks of Alzheimer’s disease (AD) pathogenesis is the accumulation of amyloid-β (Aβ) in the brain. It has been shown that this Aβ accumulation is the result of a disturbed balance of Aβ generation from its precursor APP and its subsequent clearance. In sporadic or the common late-onset AD, impaired clearance of Aβ is apparently predominantly responsible for its accumulation rather than Aβ overproduction [1]. To date, several mechanisms in the brain have been identified that are involved in the clearance of extracellular Aβ from brain interstitial fluid (ISF) and cerebrospinal fluid (CSF). These include cellular uptake followed by intracellular degradation by neurons, microglia, pericytes and astrocytes, extracellular degradation by enzymes in the ISF, efflux into the periphery by blood-brain barrier (BBB) clearance, and ISF bulk-flow clearance to the CSF sink or perivascular spaces followed by degradation or drainage into the circulatory or lymphatic system ([2, 3], reviewed in [4]). The low-density lipoprotein receptor-related protein-1 (LRP1) plays a pivotal role in Aβ clearance (reviewed in [5]) by mediating receptor-mediated Aβ uptake and degradation in astrocytes, neurons and cerebrovascular smooth muscle cells [6–8], or a concerted Aβ transcytosis together with ABCB1/P-glycoprotein (P-gp) across the BBB [9–12]. In addition, cellular models have shown that LRP1 is also a modulator of APP processing driving Aβ generation via interactions between the intracellular and extracellular domains of both transmembrane proteins LRP1 and APP [13–18]. These seemingly opposing roles of LRP1 on Aβ metabolism have raised the question to what extent LRP1 expression affects Aβ pathology in the brain.

APP is a single-pass transmembrane protein, and alternative splicing of the APP transcript generates many different isoforms of which three are most common: the 695 (APP_695_; predominantly expressed in CNS), the 751 (APP_751_), and 770 (APP_770_) amino acid forms [19]. APP isoforms encoding the Kunitz protease inhibitor domain in their extracellular part (i.e., APP_751_ and APP_770_) interact directly via this domain with a ligand-binding domain in the extracellular part of LRP1 [13, 14]. The cytoplasmic FE65 adaptor protein on the other hand can form a functional link between the intracellular domains of APP and LRP1 [15]. These interactions between LRP1 and APP allow internalization of APP into the endosomal compartment. Depending upon relative expressions levels of LRP1, of the different APP isoforms and of other involved proteins like FE65, internalization of APP and subsequent generation of Aβ can be modulated. Upon internalization of APP, amyloidogenic proteolytic cleavage by beta- and gamma-secretases results in the generation of Aβ and a soluble APP-β (sAPP-β) fragment. In contrast, non-amyloidogenic proteolytic cleavage of APP by alpha-secretase at the cell surface results in secretion of soluble APP-α (sAPP-α) and precludes generation of the detrimental Aβ peptide (reviewed in [5, 20]).

LRP1 is a single-pass transmembrane receptor that binds over 40 different ligands and is as endocytic and signaling receptor involved in many different physiological processes (reviewed in [5, 21]). After proteolytic cleavage of the LRP1 precursor, the mature LRP1 receptor consists of a large extracellular 515-kDa α-subunit (LRP1-α) non-covalently attached to its 85-kDa β-subunit (LRP1-β), consisting of an extracellular, a transmembrane, and an intracellular domain. This intracellular domain encodes many motifs, including the NPxY (xxL) motifs, interacting with adaptor and scaffold proteins. In comparison with the proximal NPxY and distal NPxY motif (within the NPxYxxL motif) and two di-leucine motifs, the YxxL motif (within the NPxYxxL motif) is apparently the predominant internalization motif in LRP1 [22]. Noteworthy is to mention that FE65 binds to the NPxYxxL motif to form a functional link between the intracellular domains of APP and LRP1, triggering the internalization of the complex and subsequent the generation of Aβ [15]. DAB1, on the other hand, competes with FE65 for LRP1 binding, resulting in a reduction of amyloidogenic APP processing [23].

Previously, we reported on the generation of a series of LRP1 knock-in mice by recombinase-mediated cassette exchange [24, 25]. In these mice, the proximal NPxY motif (also indicated by NPxY1) and/or the distal NPxYxxL motif (here indicated by NPxY2) in the intracellular domain are inactivated. The rationale to generate these mouse models was to unravel the complex biological function of the receptor LRP1 by partial impairment of the function of the endogenous LRP1 receptor. Characterization of the knock-in mouse models revealed that combined inactivation of the NPxY1 and NPxY2 motifs results in embryonic lethality between E10.5 and E13.5 reminiscent of the full LRP1 knock-out [24]. Inactivation of just NPxY1 presents late fetal lethality due to the impaired early LRP1 biosynthesis, which results in low levels of mature LRP1 reaching the cell surface [24]. Inactivation of NPxY2 (simultaneous inactivation of the overlapping NPxY and YxxL motifs) was initially not linked to a clear phenotype [25], but in a LDLR-deficient mouse model, inactivation of NPxY2 enhances postprandial dyslipidemia and atherosclerosis demonstrating that NPxY2 is essential for the atheroprotective role of LRP1 [26]. Furthermore, NPxY2 inactivation was shown in vitro in mouse-derived cells to compromise LRP1 endocytosis rates of ApoE, α_2_M and NR2B NMDA receptor subtype, and β1-integrin [24, 27–29]. This is mainly a consequence of inefficient slow recycling of the mutated receptor [27]. We also identified the NPxY2 motif of LRP1 as a crucial element for LRP1-NMDA receptor interaction via the adaptor protein PSD95 and relevant for tPA activation of the LRP1-NMDA receptor complex in derived neuronal cells [30]. Finally, inactivation of NPxY2 resulted in impairment of transcytosis of Aβ1–40 across a brain endothelial monolayer [10].

In the current study, we sought to study the dual role of LRP1 on Aβ metabolism in vivo. We report on the in vivo impact of the LRP1-inactivating NPxY2 mutation on APP processing in a mouse model overexpressing human APP. Our results provide the first in vivo evidence that in accordance with the cellular in vitro models, endogenous LRP1 contributes to the generation of Aβ and simultaneously is a receptor for Aβ clearance. A loss of LRP1 function results in a reduction of Aβ clearance, but at the same time favors non-amyloidogenic APP processing, reducing the overall generation of Aβ and leading to a diminished Aβ pathology in brain.

## Methods

### Mice

Generation of a LRP1-NPxY2 mutant knock-in mouse with inactivation of the membrane distal NPxY2 (NPVYATL ➔ AAVAATL) motif was already described in detail before [25]. For analysis of the in vivo effect of inactivation of the membrane distal NPxY2 motif on the APP metabolism in a AD mouse model, LRP1-NPxY2 mutant mice were crossed with the Thy1-hAPP_751_SL AD mouse model described by Blanchard et al. [31], overexpressing a human APP_751_ isoform carrying the Swedish and London mutations. Breeding pairs of heterozygous LRP1-NPxY2 mutant knock-in mice of which the male or female was additionally carrying the Thy1-hAPP_751_SL transgene were used to generate inbred LRP1 wildtype mice and homozygous LRP1-NPxY2 mutant knock-in mice, without or hemizygous for the Thy1-hAPP_751_SL transgene (mixed C57Bl/6J and 129 background). Unless otherwise stated, female mice of an age of 9 months were analyzed in a comparative study. For in vitro endothelial transcytosis analysis, inducible brain endothelial-specific LRP1 knock-out mice (*Lrp1*_BE_^−/−^) were used as described in detail before [12]. The research was approved by the Ethical Committees for Animal Experimentation of the KU Leuven and the Johannes Gutenberg University of Mainz and the ethical committee on animal care and use of Rhineland-Palatinate, Germany.

### Isolation of Total Protein Homogenates and Protein Fractionation from Mice Brains

Mice, euthanized by CO_2_ intoxication or pentobarbital (100 mg/kg, i.p.), were perfused transcardically with PBS. After isolations of the brains, hemispheres were snap-frozen in liquid nitrogen and stored at − 80 °C until further processing. For the analysis of APP metabolism in protein subfractions, 6-ml ice-cold buffer [20-mM Tris-HCl, 1× complete proteinase cocktail (Roche) (pH 8.5)] per gram frozen tissue was used for homogenization with a cooled glass-Teflon Potter-Elvehjem type of homogenizer (18 strokes, 350 rpm). Total protein homogenates were obtained after brief centrifugation (12,000 *g*, 15 min, 4 °C) to remove debris. Small aliquots of the total protein homogenates were stored at − 20 °C, whereas the remaining large aliquots were used according to a differential extraction procedure to obtain fractions containing soluble proteins, TX100-soluble membrane proteins, and insoluble or “plaques”-associated proteins as described before with minor modifications [32]. After an initial centrifugation step (100,000 ×*g* for 80 min at 4 °C, Beckman TL100), the supernatant was collected as soluble protein fraction, whereas the pellet fraction was resuspended in 6-ml ice-cold buffer supplemented with 1% TX100 per gram pellet fraction. After a next centrifugation step (100,000 ×*g* for 80 min at 4 °C, Beckman TL100), the supernatant was collected as the TX100-soluble membrane protein fraction. All fractions were stored at − 20 °C until analysis. For analysis of the different Aβ species in PBS-soluble protein fractions by a combination of immunoprecipitation and western blot analysis, a different fractionation protocol was used [12]. Briefly, brain hemispheres were homogenized in 1000-μL PBS containing complete protease and phosphatase inhibitor (Roche Applied Science) using a glass homogenizer (30 strokes) and subsequently centrifuged at 55,000 *g* for 20 min at 4 °C. The supernatant containing secreted PBS-soluble brain Aβ was collected and stored at − 20 °C for further analysis.

### Western Blot Analysis of APP and APP Metabolites and ELISA for Aβ

Protein concentrations of the soluble protein fractions and of the TX100-soluble membrane protein fractions were determined by the bicinchoninic acid assay kit (PIERCE, Perbio, France), and samples containing 10-μg protein were prepared in LDS sample buffer (Invitrogen NP-009). After reduction and denaturation at 95 °C for 10 min, the samples were loaded and separated by PAGE on 4–20% or 10% Tris-glycine (Anamed, Germany) and transferred onto nitrocellulose membranes. Ponceau S (Sigma-Aldrich) was used to confirm loading of equal amounts of protein and to monitor the transfer procedure. After blocking with blocking buffer [TBS (50-mM Tris, 150-mM NaCl, pH 7.6) containing 0.1% Tween 20 and 5% milk], the membranes were probed overnight (4 °C) with a primary antibody diluted in blocking buffer. Membranes were rinsed in TBS containing 0.1% Tween 20 and incubated with appropriate horseradish peroxidase (HRP)-conjugated secondary antibodies diluted in blocking buffer. Careful execution of the homogenization and fractionation procedures in combination with the Ponceau S staining of the blots confirmed loading and subsequent transfer of equal amounts of protein in the analyzed protein fractions (see Supplementary Fig. 1a, Supplementary Fig. 2a, b). In the case of western blot analysis of total homogenates, usually, normalization of western blot expression levels to housekeeping genes, like actin or tubulin, is done. At first sight, use of analysis of actin expression in combination with a fractionation procedure following upon homogenization appears less suitable, but in our fractionation procedure the distribution of actin between the soluble protein fraction and TX100-soluble membrane protein fractions was shown to occur according a relatively stable and reproducible ratio (see Supplementary Fig. 1b), confirming also in this way loading and transfer of equal amount of protein in the analyzed protein fractions. Moreover, we analyzed up to *n* = 5–6 animals per group to get representative results.

For detection of immature and mature APP in the TX100-soluble membrane protein fraction, the primary rabbit polyclonal antibody B10.4 (homemade, directed against the 20 carboxy-terminal amino acids of APP) was used. For detection of total sAPP and sAPP-α in the soluble protein fraction, the primary mouse monoclonal antibodies 22C11 (Chemicon, directed against aa 6–81 of APP) and 6E10 (Chemicon, directed against aa 1–17 of Aβ) were used respectively. For detection of sAPP-β in the soluble protein fraction, a rabbit antibody (Signet, 9138-005) was used. Mouse anti-tubulin (T8328, Sigma-Aldrich), mouse anti-synaptophysin (101011, Synaptic Systems), and mouse anti-PSD-95 (P43520, BD Transduction Laboratories) were used to control for equal loading of membrane fractions. Finally, mouse monoclonal anti-β-actin clone AC-15 (Sigma-Aldrich) was used for analysis of actin expression. Secondary antibodies were conjugated with HRP. Western blots were developed by chemiluminescence (Western Lightning ECL Pro; Perkin-Elmer) followed by digital picture acquisition and analysis (LAS 4000; ImageQuant v7.0; GE Healthcare). Final blot pictures were equally adjusted to enhance visibility using Adobe Photoshop (version 7.0) (Adobe Systems, San Jose, USA).

### Immunoprecipitation of Total Aβ from PBS-Soluble Protein Fractions and Subsequent Aβ Separation with 8-M Urea SDS Gel and Western Blotting

Total Aβ was immunoprecipitated from 1000-μg protein of PBS-soluble protein fractions by mixing fivefold concentrated detergent buffer [50-mM HEPES (pH 7.4), 150-mM NaCl, 0.5% (*v*/*v*) Nonidet P-40, 0.05% (*w*/*v*) SDS, and protease inhibitor cocktail (Roche Applied Science)] with the respective samples. Magnetic Dynabeads (M-280 Sheep Anti-Mouse IgG, 11201D, Novex) containing sheep anti-mouse IgG attached to their surface were precoated with IC16 antibody directed against the first 16 amino acids of Aβ [33] according to the manufacturer’s protocols, and equal amounts of IC16 antibody-covered beads were added to the samples as shown by the immunoreactivity of IC16 light chain displayed in Supplementary Fig. 3. After overnight incubation at 4 °C, samples were washed 3 times in PBS, 0.1% (*w*/*v*) BSA, and once in 10-mM Tris-HCl, pH 7.5. After heating the samples to 95 °C in 25-μl sample buffer [0.36-M Bis-Tris, 0.16-M bicine, 1% (*w*/*v*) SDS, 15% (*w*/*v*) sucrose, and 0.0075% (*w*/*v*) bromphenol blue], the supernatants were subjected to PAGE. Separation of immunoprecipitated Aβ peptides was performed on 0.75-mm 10% T/5% C polyacrylamide 8-M urea SDS gels. For separation of Aβ1–40 from Aβ1–42, a final concentration of 0.3-M H_2_SO_4_ was used in resolving gels. This has the effect that peptides are separated not only according to their molecular weight but also according to their hydrophobicity and results that Aβ1–42 migrates faster than Aβ1–40. Peptides were transferred to an Immobilion-P PVDF membrane via semi-dry western blotting (Bio-Rad) at 46 mA for 45 min. Membranes were boiled for 3 min in PBS and blocked in 5% skim milk in TBST [20-mM Tris, 137-mM NaCl, 0.1% (*v*/*v*) Tween-20] for 30 min afterward. Aβ peptides were detected with IC16 antibody and donkey anti-mouse second antibody conjugated with HRP using enhanced chemiluminescence (Millipore, Schwalbach, Germany) and digital picture acquisition (LAS-3000mini, Fujifilm, Duesseldorf, Germany).

### Isolation of Cerebrospinal Fluid for Aβ ELISA

For isolation of CSF from the cisterna magna, the method previously described by DeMattos was used [34]. Subsequently, the Aβ40 and Aβ42 levels in the CSF were determined by Aβ40 and Aβ42 specific ELISA kits, according to the manufacturer’s instructions (the Genetics Company).

### Isolation and Culture of Primary Mouse Brain Capillary Endothelial Cells

Primary mouse brain capillary endothelial cells were isolated from 12- to 15-week-old mice according to a standard protocol as described previously [10, 35]. Cells were plated on 24-well Transwell filters (pore size, 0.4 μm; surface area, 33.6 mm^2^; Greiner Bio-One) coated with collagen IV/fibronectin (Sigma-Aldrich). Cultures were maintained in DMEM supplemented with 20% plasma-derived bovine serum (First Link), 100-U/ml penicillin (Gibco), 100-μg/ml streptomycin (Gibco), 2-mM L-glutamine (Gibco), 4-μg/ml puromycin (Alexis), and endothelial cell growth supplement (E2759, Sigma) at 37 °C and 5% CO_2_. Cells were cultured in the cellZscope device, in which transendothelial electrical resistance (TEER) and capacitance were monitored over time. Puromycin was withdrawn after 4 days in culture. When cells reached confluency and the capacitance was around 1 μF/cm^2^, culture medium was removed, and serum-free DMEM/Ham’s F12 (Gibco) medium containing 1-mM L-glutamine, 100-U/ml penicillin, and 100-μg/ml streptomycin was added. Five hundred fifty nM hydrocortisone (Sigma-Aldrich) was supplemented to induce high TEER. The following day transport studies were performed.

### In Vitro Transcytosis of [^125^I]-Aβ1–42

In order to study Aβ transcytosis in vitro, a standard transport model was used [10, 12, 35]. [^125^I]-Aβ1–42 (0.1 nM) (purchased from Phoenix Peptide) and 1-μCi/ml [^14^C] inulin (purchased from PerkinElmer), a marker for paracellular diffusion, were added to serum-free media supplemented with 550-nM hydrocortisone and 40-mM HEPES and incubated at 37 °C. To study brain-to-blood transport, 10- and 80-μl samples were taken from the luminal compartment 60 min after the addition of [^125^I]-Aβ1–42 and [^14^C]-inulin to the abluminal compartment. To investigate the amount of intact [^125^I]-Aβ1–42 transported to the luminal side, 80-μl 15% TCA was added to an 80-μl luminal media sample and incubated for 10 min at 4 °C. Samples were then centrifuged at 16,000 *g* for 10 min. Pellets (representing intact [^125^I]-Aβ1–42) were counted for [^125^I]. Probes were counted on a Wallac Wizard2 2470 automatic γ-counter (PerkinElmer) for [^125^I] or on a Tri-Carb 2800 TR Liquid Scintillation Analyzer (PerkinElmer) for [^14^C]. Transport of intact [^125^I]-Aβ1–42 across the monolayer was calculated as Aβ1–42 transcytosis quotient (TQ) using the following formula:

$$ \mathrm{A}\upbeta 1-42\ \mathrm{TQ}=\left(\left[{}^{125}\mathrm{I}\right]-\mathrm{A}\upbeta 1-42\ \mathrm{luminal}/\left[{}^{125}\mathrm{I}\right]-\mathrm{A}\upbeta 1-42\ \mathrm{input}\right)/\left(\left[{}^{14}\mathrm{C}\right]-\mathrm{inulin}\ \mathrm{acceptor}/\left[{}^{14}\mathrm{C}\right]-\mathrm{inulin}\ \mathrm{input}\right) $$

### Brain Clearance of [^125^I]-Aβ1–42

To measure the brain clearance of [^125^I]-Aβ1–42, we used a similar method as described previously [12]. 0.5-μl tracer fluid containing 1 μCi/ml [^14^C]-inulin (reference marker) and pathophysiological amounts of [^125^I]-Aβ1–42 (5.14 nM) was injected with a 26-gauge needle attached to an UltraMicroPump controller (UMP3-1, Word Precision Instruments) over 5 min into ISF of the right caudate putamen (0.9 mm anterior from bregma, 1.9 mm lateral from midline, and 2.9 mm below the surface) of 4 months old and sex-matched mice anesthetized with ketamine (800 mg/kg) and medetomidine (5 mg/kg). After injection, the microsyringe was left in place for 5 min. Blinded brain samples were collected at 10 min after injection and prepared for analysis. In vivo [^125^I]-Aβ1–42 clearance was calculated as follows: 100% recovery in brain after 15 min. The percentage of radioactivity remaining in the brain was calculated as follows: % recovery in brain = 100 × (*N*_b_ / *N*_i_), where *N*_b_ is the radioactivity in the brain at the end of the experiment and *N*_i_ is the radioactivity injected into the brain, as illustrated by TCA-precipitable [^125^I]-radioactivity (measured in cpm).

### Immunohistochemistry

Mice, euthanized by CO_2_ intoxication, were perfused transcardically with PBS followed by a 4% paraformaldehyde (PFH) solution in phosphate-buffered saline (PBS). After isolation of the brains, brains were postfixated overnight in 4% PFH in PBS and finally rinsed in PBS. The brains were then dehydrated and embedded in paraffin for sectioning (7 μm in thickness). Paraffin-embedded brain sections were dewaxed and rehydrated, the epitope was exposed in 1/3 formic acid for 7 min, and endogenous peroxidase was quenched with hydrogen peroxide [3% (*v*/*v*) in methanol] for 10 min. After sufficient washing with PBS, slides were incubated for 5 min in PBS supplemented with 1% Tween 20 followed by 2 h in a blocking buffer [5% goat normal serum, 2% BSA, 0.5% blocking reagent (Perkin Elmer), 10-mM Tris-HCl, 150-mM NaCl, pH 7.5]. The primary antibody diluted in blocking buffer was applied overnight at 4 °C. Following sufficient washing, secondary antibody diluted in blocking buffer was applied for 2 h at RT. For analysis of the plaque load, primary mouse monoclonal antibody 6E10 (Chemicon, directed against amino acids 1–17 of Aβ) and secondary antibody GAM-HRP were used, and the signal was visualized with the TSA™ PLUS kit from Perkin Elmer according to the manufacturer’s instructions. Photographs were taken with a Leica MZ FLIII stereomicroscope. The number of plaques and the percentage of the brain covered with plaques was analyzed by ImageJ 1.41 software from the National Institute of Health (NIH) on four consecutive sections from each mouse around bregma − 2.5.

### Statistical Analysis

Statistical significance was assessed using Student’s *t* test. Whenever necessary, data were logarithmically transformed to meet *t* test assumptions. *p* < 0.05 was regarded statistically significant.

## Results

### Decreased Brain Clearance of Aβ in Mice Carrying the NPxY2 Knock-in Mutation

**Fig. 1:**
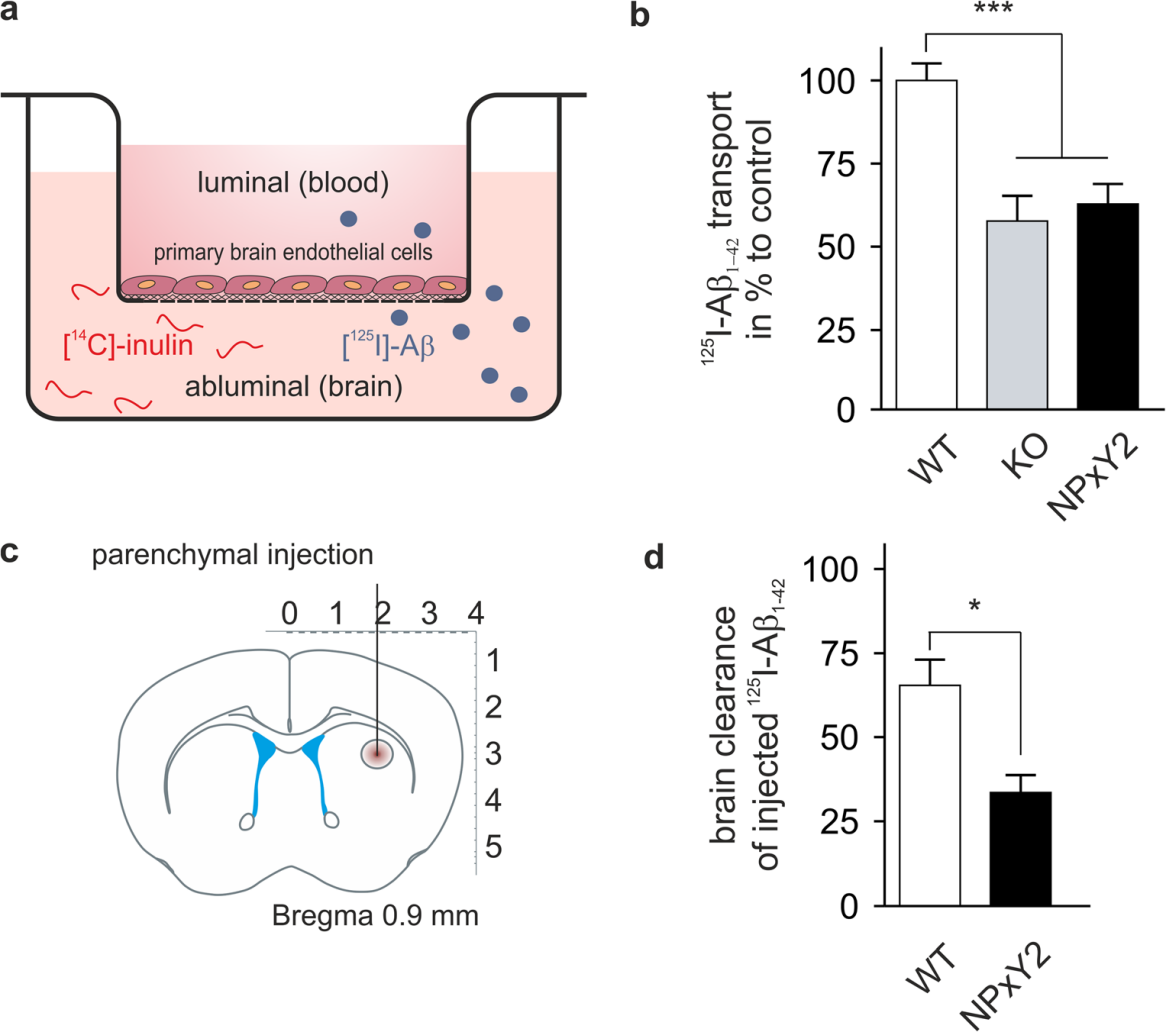
Decreased Aβ brain clearance and endothelial transcytosis in LRP1 NPxY2 mice. **a** Diagram illustrating the experimental procedure of [^125^I]-Aβ1–42 transport across the primary mouse brain capillary endothelial cell monolayer cultured on Transwell inserts. **b** [^125^I]-Aβ1–42 transport across the primary mouse brain capillary endothelial cell monolayer derived from control LRP1^WT/WT^ (WT), LRP1_BE_^−/−^ (brain endothelial-specific knock-out, KO), and LRP1^NPxY2/NPxY2^ (NPxY2) mice was studied in the presence of 1-μCi/ml [14C]-inulin to determine the transcytosis quotient (TQ). Transcytosis was analyzed in the brain-to-blood direction (abluminal to luminal) by measuring the dpm for [^14^C]-inulin and the cpm for the TCA-precipitable [^125^I] radioactivity. Data of three independent experiments, *n* = 22 (WT), *n* = 12 (KO), and *n* = 17 (NPxY2) (age 12–15 weeks). **c** Diagram illustrating the experimental procedure of in vivo brain clearance in mice. **d** LRP1 NPxY2 knock-in mutation inhibits Aβ brain clearance in vivo in LRP1^NPxY2/NPxY2^ (NPxY2) mice compared with control LRP1^WT/WT^ (WT) mice. 5.14-nM [^125^I]-Aβ1–42 was microinfused into brain ISF of the caudate nucleus. Efflux was studied 15 min after injection by determining remaining radioactivity in the brain. *n* = 4 (WT and NPxY2, age 4 months). Error bars represent SEM. For statistical analyses of the data in B, the following test was used: repeated-measures ANOVA followed by Bonferroni multiple comparisons. For statistical analyses of the data in **d**, Student’s *t* test was used. * indicates *p* < 0.05, *** indicates *p* < 0.001

Previously, we have shown that primary endothelial cells derived from LRP1^NPxY2/NPxY2^ mice show reduced transport of Aβ across the endothelial cells compared with those from LRP1^WT/WT^ mice, corroborating that LRP1 is an important clearance receptor for neurotoxic Aβ from brain [9, 10]. In our present analysis, we verified our original findings (Fig. 1a, b), showing that transcytosis of Aβ1–42 was reduced in endothelial cells carrying the LRP1 NPxY2 knock-in mutation compared to wild-type (wt) endothelial cells. The reduction in transport we observed was comparable to endothelial cells deficient of LRP1 (KO) implicating that Aβ transport capacity is highly impaired by the NPxY2 knock-in mutation [12]. These data again demonstrated that on a cellular level, impaired LRP1 function due to its decreased internalization leads to decreased Aβ clearance. LRP1 expression is not restricted to cells compromising the BBB. In fact, it has been shown that also neuronal, astrocytic LRP1, and also LRP1 in smooth muscle cells contribute to the clearance of Aβ in vivo [6, 7, 36]. In order to study total brain clearance in vivo, we injected radiolabelled [^125^I]-Aβ1–42 into the caudate putamen of LRP1^NPxY2/NPxY2^ and LRP1^WT/WT^ mice (Fig. 1c). Whereas Aβ was rapidly cleared from brain in LRP1^WT/WT^ mice, LRP1^NPxY2/NPxY2^ showed a significant impairment in Aβ clearance from brain (Fig. 1d). Together, these results demonstrated that not only on a cellular level but also in vivo LRP1^NPxY2/NPxY2^ mice show a reduction in the capacity of Aβ clearance.

**Fig. 2:**
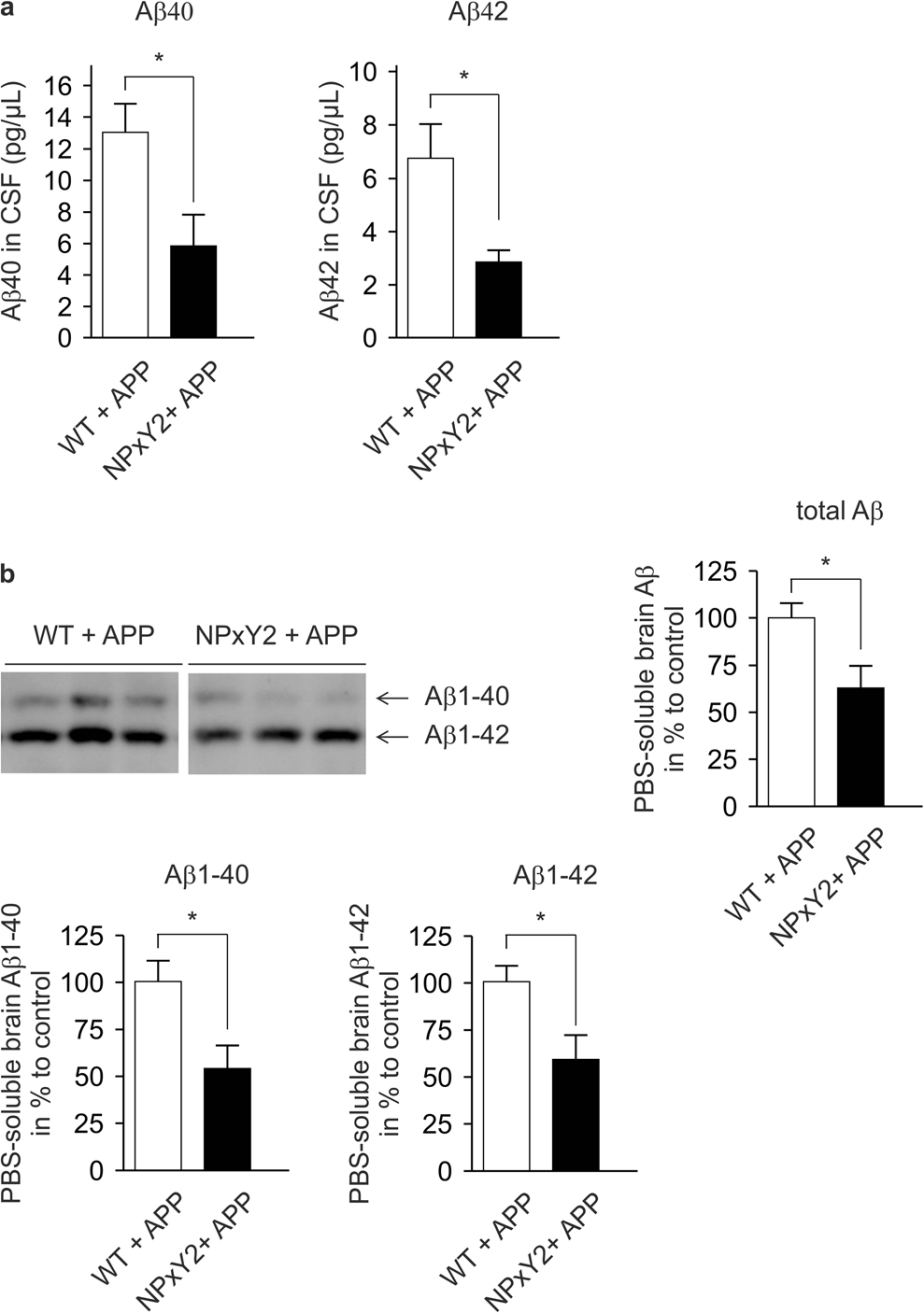
Decreased levels of Aβ in brain fluids of LRP1 NPxY2 mice. **a** ELISA on CSF obtained from the cisterna magna showed significant reduced levels for Aβ40 and Aβ42 in LRP1^NPxY2/NPxY2^/hAPP_751_SL (NPxY2 + APP) mice compared with LRP1^WT/WT^/hAPP_751_SL (WT + APP) mice. *n* = 10 for both groups (age 9 months). **b** WB analysis of the PBS-soluble protein fractions showed significant reductions in total Aβ, Aβ1–42, and Aβ1–40 levels in the NPxY2 + APP mice compared to control WT + APP. *n* = 5 (WT + APP) and *n* = 6 (NPxY2 + APP) (age 9 months). Error bars represent SEM, and statistical analysis was performed with Student’s *t* test, * indicates *p* < 0.05

As we saw that NPxY2-mutated LRP1 showed a reduction in Aβ clearance in vitro and in vivo, we crossed LRP1^NPxY2/NPxY2^ mice with Thy1-hAPP_751_SL mouse model that expresses human APP bearing both the Swedish (K670N/M671L) and the London (V717I) mutations (LRP1^NPxY2/NPxY2^/hAPP_751_SL). We analyzed the CSF of 9-month-old mice by Aβ ELISA measures. Interestingly, we found that CSF concentrations of both Aβ40 and Aβ42 in LRP1^NPxY2/NPxY2^/hAPP_751_SL were significantly lower than in LRP1^WT/WT^/hAPP_751_SL control mice (Fig. 2a). This finding seemed to contradict our original idea that Aβ should accumulate due to impaired brain clearance. Moreover, we analyzed the soluble levels of Aβ in the brain by homogenizing the brain hemispheres in PBS followed by immunoprecipitation. Corresponding to what we saw in the CSF, we found significantly lower Aβ1–40 and Aβ1–42 levels in the PBS-soluble fraction of the brain as analyzed by SDS-urea gel electrophoresis (Fig. 2b). Although several measures showed lower Aβ levels in fluids of the CNS in mice with impaired LRP1 function, it seemed to contradict all findings from previous studies showing that LRP1 impairment results in the accumulation of Aβ due to impaired clearance [11, 12, 37].

**Fig. 3:**
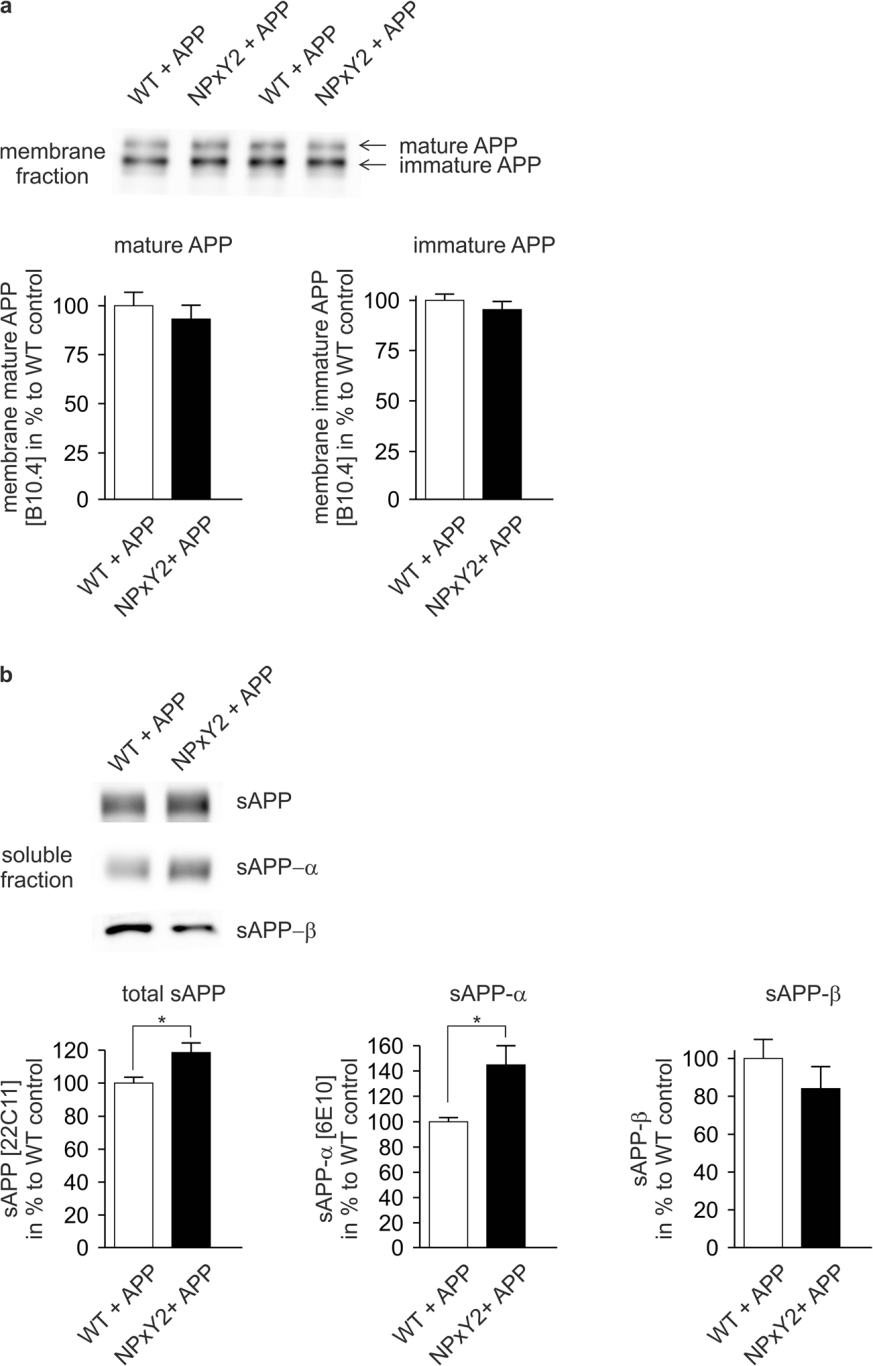
No difference in APP expression but difference in sAPP shedding in LRP1 NPxY2 knock-in mice. **a** WB analysis of the TX100-soluble membrane protein fractions showed no differences in the expression levels of unprocessed full length immature and mature APP in brains of LRP1^NPxY2/NPxY2^/hAPP_751_SL (NPxY2 + APP) mice compared with control LRP1^WT/WT^/hAPP_751_SL (WT + APP) mice. **b** WB analysis of the soluble protein fractions revealed a significant increase of total sAPP and sAPP-α in LRP1^NPxY2/NPxY2^/hAPP_751_SL (NPxY2 + APP) mice compared with control LRP1^WT/WT^/hAPP_751_SL (WT + APP) mice. The trend in reduction in sAPP-β did not reach statistical significance. Number of mice analyzed: *n* = 6 mice (WT + APP) and 5–6 (NPxY2 + APP) (age 9 months). Error bars represent SEM, and statistical analysis was performed with Student’s *t* test, * indicates *p* < 0.05

Next, we analyzed APP expression in brains of LRP1^NPxY2/NPxY2^/hAPP_751_SL and LRP1^WT/WT^/hAPP_751_SL controls with western blot analysis after protein fractionation. Loading and subsequent transfer of equal amounts of protein in the analyzed protein fractions were confirmed by Ponceau S staining and analysis of the expression of actin, which was apparently distributed according to a reproducible and stable ratio between the soluble protein fractions and TX100-soluble membrane protein fractions (see Supplementary Fig. 1a, b). Additionally, we analyzed membrane proteins PSD-95 and synaptophysin as an internal control for equal loading of membrane fractions (see Supplementary Fig. 2a, b). We could not see any effects on the total mature an immature forms of APP in the TX100-soluble membrane protein fraction (Fig. 3a), indicating that no differences in APP expression are the reason for the reduced levels of Aβ found in CSF and PBS-soluble fractions. Analysis of the soluble protein fraction, however, showed that of LRP1^NPxY2/NPxY2^/hAPP_751_SL exhibited in significant increase of total sAPP and sAPP-α levels (Fig. 3b), suggesting that APP processing is altered in these mice. Vice versa, we could detect a trend towards lower sAPP-β levels that did not reach statistical significance (Fig. 3b).

The evident increase in sAPP-α and reduction in soluble total Aβ indicated that inactivation of LRP1 through its NPxY2 knock-in mutation shifts APP from amyloidogenic by beta-secratase to non-amyloidogenic processing by alpha-secretase at the cell surface, presumably due to impaired APP endocytosis by LRP1.

### Inactivation of the NPxY2 Motif Is Associated with a Reduced Aβ Plaque Load in Brains of APP_751_SL Mice

As our findings suggested that reduced endocytosis of APP by the impaired function of LRP1 promotes APP processing by alpha-secretase and thus lowering the generation of Aβ, we analyzed the plaque deposition in aged mice overexpressing human APP (hAPP_751_SL). Female mice were used, because they show an earlier phenotype than males.

**Fig. 4:**
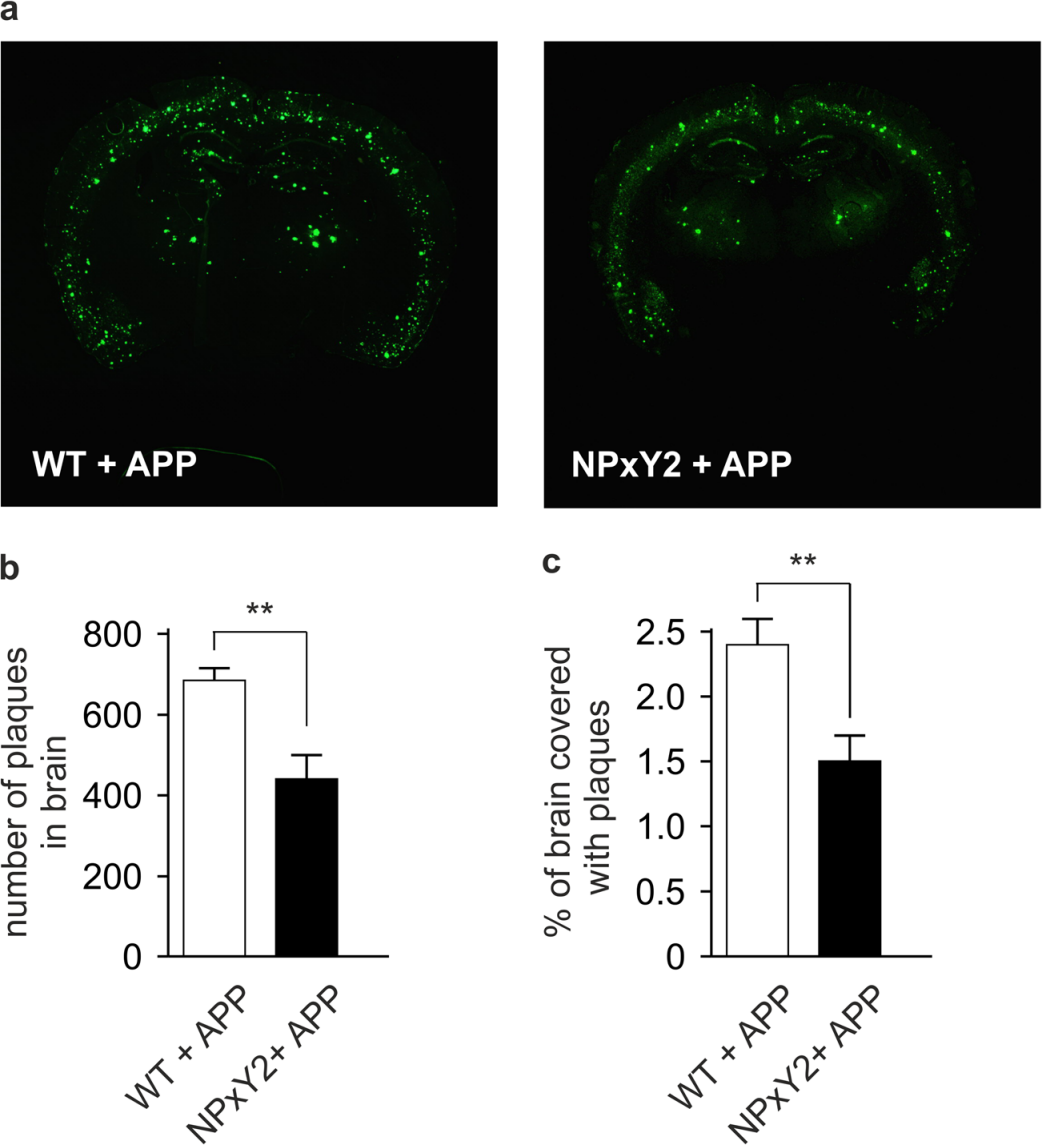
Plaque load in brain is significantly reduced in LRP1 NPxY2 mice. **a** Brain slices (bregma − 2.5) of LRP1^WT/WT^/hAPP_751_SL (WT + APP) and LRP1^NPxY2/NPxY2^/hAPP_751_SL (NPxY2 + APP) mice were immunostained with the antibody 6E10, and plaques were visualized with the TSA™PLUS kit from Perkin Elmer. Pictures show representative stainings for WT + APP and NPxY2 + APP mice. **b** The number of plaques as determined in 6E10-immunostained brain slices via ImageJ software from NIH. **c** The percentage of the brain covered with plaques as determined in 6E10-immunostained brain slices via ImageJ software from NIH. For **b** and **c**, *n* = 10 for LRP1^WT/WT^/hAPP_751_SL (WT + APP) mice and *n* = 8 for and LRP1^NPxY2/NPxY2^/hAPP_751_SL (NPxY2 + APP) mice (age 9 months). Error bars represent SEM, and statistical analysis was performed with Student’s *t* test, * indicates *p* < 0.05

Brain sections of 9-month-old female LRP1^WT/WT^/hAPP_751_SL and LRP1^NPxY2/NPxY2^/hAPP_751_SL mice were immunostained for Aβ (6E10 antibody) to visualize plaques. Analyses of four consecutive brain slices confirmed that the total number of plaques in the brain (Fig. 4a, b) as well as the percentage of the brain covered with plaques (Fig. 4a, c) was significantly reduced by 36 and 38% in the LRP1^NPxY2/NPxY2^/hAPP_751_SL mice when compared with the LRP1^WT/WT^/hAPP_751_SL controls. Therefore, inactivation of LRP1 does not only affect the soluble brain levels of Aβ but also influence its plaque deposition despite impaired Aβ clearance of LRP1.

## Discussion

The objective of the present study was to investigate in vivo the impact of impaired LRP1 function on the APP processing. Previously, it has been shown that inactivation of the NPxY2 motif of endogenous LRP1 results in a partial inhibition of the endocytosis (and transcytosis in brain endothelial cells) of ligands or complexes with co-receptors by this LRP1 receptor [10, 24, 26–30]. In the present study, we could confirm that impairment of LRP1 endocytosis inhibits the brain clearance of Aβ in vitro and in vivo. At the same time, we found that impairment of LRP1 endocytosis influences the overall generation of Aβ by promoting alpha-secretase cleavage at the cell surface (as seen by higher sAPP-α levels) and the prevention of Aβ generation after endocytosis in vivo. Interestingly, our collective results suggest that despite impaired Aβ brain clearance due to the inactivation of LRP1, there is less accumulation of Aβ in CSF, ISF, and in plaques in a mouse model of AD in vivo.

This study clearly demonstrates that LRP1 has a dual role APP metabolism affecting both generation and clearance of Aβ. Many studies have shown both in vitro and in vivo that LRP1 is an important mediator in the clearance of Aβ from the brain, e.g., in receptor-mediated endocytosis in astrocytes, neurons, and cerebrovascular smooth muscle cells [6–8]; in transcytosis across the BBB [9–12]. Cell type-specific deletion of LRP1 in astrocytes, neurons, smooth muscle cells, and endothelial cells has all led to a general increase of brain Aβ. Studies on the general impact of LRP1 in different cell types on Aβ metabolism or its effect on APP processing, and thus, the generation of Aβ in vivo is scarce. Many in vitro studies support a role for LRP1 in the generation of Aβ [13–18]. However, direct in vivo support for the involvement of LRP1 is limited. It has been shown that overexpression of a functional LRP1 mini-receptor in an APP transgenic mouse model [38] resulted in an age-dependent increase of soluble brain Aβ [39]. These observations in this overexpressing mouse model support our findings and LRP1’s role in amyloidogenic processing of APP in vivo. This study, for the first time, has modulated endogenous and global LRP1 function and has studied the effect on Aβ metabolism. Overall inhibition of endogenous LRP1 is expected to simultaneously affect generation and clearance of Aβ, in which opposing effects could mask each other. In our present study, we see both: due to LRP1 inactivation in vivo brain clearance and, at the same time, the generation of Aβ is impaired. Still, the net effect of impaired LRP1 function on brain Aβ, despite its impaired clearance, is a lesser accumulation of Aβ in the brain.

**Fig. 5:**
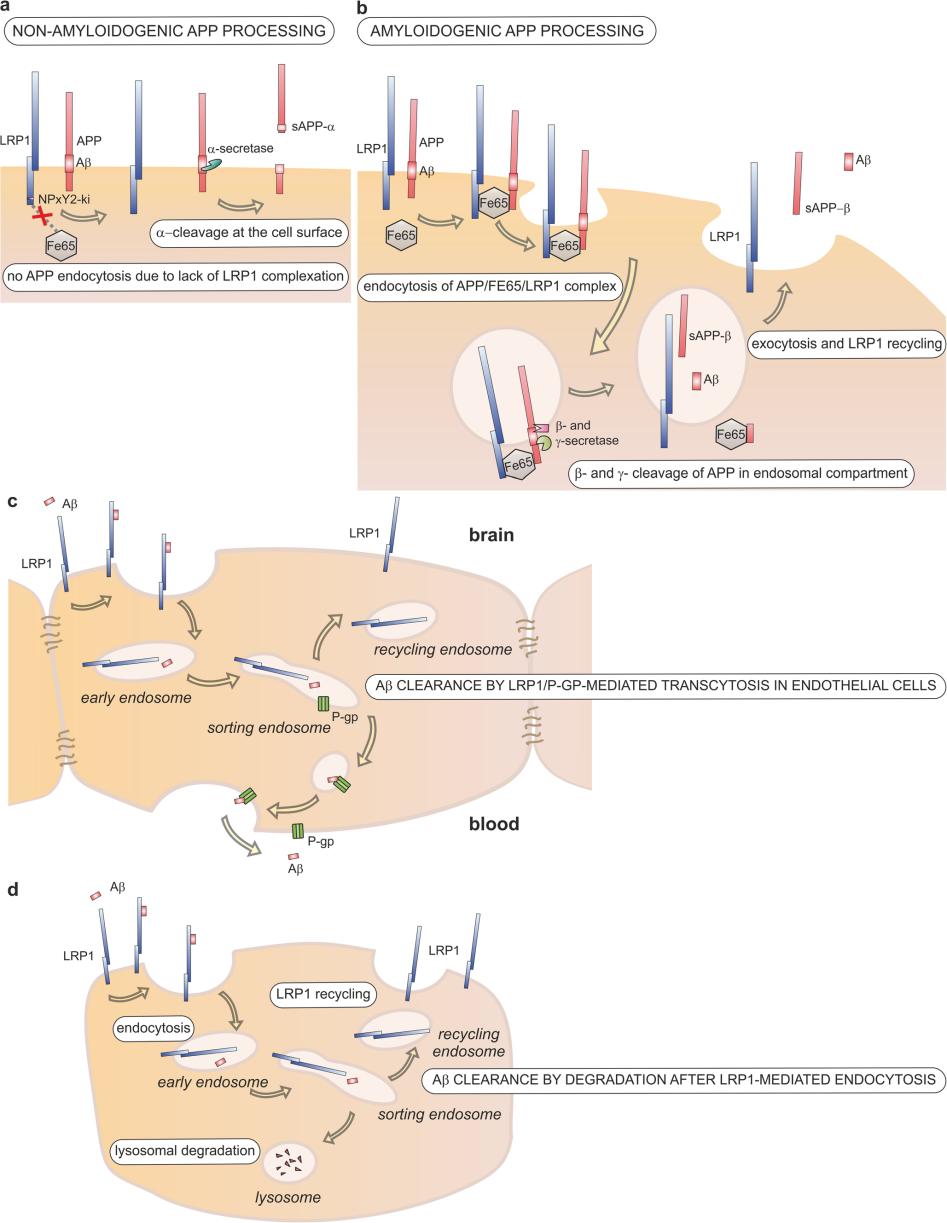
The role of LRP1 in APP processing and Aβ clearance in the brain. **a** Schematic representation of non-amyloidogenic processing of APP: α-cleavage at the cell surface by α-secretase precludes generation of Aβ and results in generation of sAPP-α. The lack of FE65 binding to the LRP1 NPxY2 domain due to the knock-in mutation reduces APP internalization. **b** Schematic representation of amyloidogenic processing of APP: LRP1 mediates the internalization of the APP/LRP1/FE65 complex, which results in the intracellular generation of Aβ. Interaction between FE65 and the intracellular domains of APP and LRP1 triggers the internalization of the tripartite complex. Subsequent β- and γ-cleavage by β- and γ-secretases in the endosomal compartment results in the generation of Aβ and sAPP-β, which are both secreted by exocytosis, a process that simultaneously results in the recycling of LRP1 to the cell surface. **c** Involvement of LRP1 Aβ clearance from brain ISF in the brain by endothelial transcytosis across the blood-brain barrier. In ISF-circulating Aβ is internalized by LRP1 and handed over to ABCB1/P-glycoprotein (P-gp) probably in the sorting endosome. P-gp exocytoses Aβ at the luminal side of the endothelium, whereas LRP1 is recycled to the cell surface. **d** Endocytosis by astrocytes, neurons, smooth muscle cells, and other cells in the brain results in lysosomal degradation of Aβ

Thus, the present results are a direct in vivo support that LRP1 is involved in amyloidogenic APP processing. Hence, the receptor that is involved in the generation of potentially toxic Aβ through receptor-mediated endocytosis of APP, at the same time, keeps soluble brain Aβ low by the constant clearance of Aβ from ISF and CSF through endocytosis and subsequent degradation or transcytosis across the BBB (see Fig. 5). The clearance function of LRP1 in the different cells of the brain seems crucial as multiple cell-specific knock-out mouse models have shown that LRP1 ablation leads to a net accumulation of Aβ in the brain. One has to say that this carefully balanced system of Aβ production and clearance is influenced not only by LRP1 expression itself but also by other members of the LDLR family that modulate APP endocytosis (reviewed in [40]). Therefore, changes in expression of different LDLR family members will modulate the generation versus clearance machinery. However, our study concentrating on LPR1 function on APP metabolism in vivo suggests that LRP1 is actually promoting the generation of Aβ, while at the same time preventing its accumulation by mediating its clearance.

It is noteworthy to say that it is most likely not feasible to target LRP1 expression in the brain to reduce the production of Aβ: LRP1 has numerous other functions besides mediating endocytosis. Over the years, studies have shown that LRP1 is involved in many important cellular processes, including cell signaling, cell migration, proliferation, angiogenesis, and wnt signaling modulation ([41], reviewed in [42]). Embryonic lethality of global LRP1 knock-out mice shows the importance of the receptor in these processes during development. On the other hand, cell-specific LRP1 knock-out mouse models have shown that clearance of Aβ can be modulated by LRP1 expression [6, 7, 12, 36]. As it is unlikely that we can control LRP1-mediated production of Aβ, future therapeutic approaches should concentrate on enhancing LRP1-mediated Aβ clearance from the brain.

In conclusion, our findings for the first time show that endogenous LRP1 has a predominate role in regulating the processing of APP in vivo. Our analysis in a mouse model of AD shows that impairment of LRP1 inhibits Aβ brain clearance but simultaneously reduces its production resulting in an overall reduced Aβ pathology in the brain and therefore gives important insights in the molecular mechanisms underlying metabolism of Aβ in vivo.

## Electronic Supplementary Material

ESM 1 (DOCX 1.68 mb)
