# Supplementary material for: LRP1 Has a Predominant Role in Production over Clearance of Aβ in a Mouse Model of Alzheimer’s Disease
Source: Mol Neurobiol. 2019 Apr 19;56(10):7234–45. doi: 10.1007/s12035-019-1594-2 (PMC6728278; doi:10.1007/s12035-019-1594-2)
Supplement: Supplementary file 1 — (DOCX 1.68 mb) [file 12035_2019_1594_MOESM1_ESM.docx]

**Supplementary material**

(supplementary figures with legends)

Corresponding to

**LRP1 has a predominant role in production over clearance of Aβ in a mouse model of Alzheimer’s disease**

by

Bart Van Gool, Steffen E. Storck, Sara M. Reekmans, Benoit Lechat, Philip L.S.M. Gordts, Laurent Pradier, Claus U. Pietrzik, Anton J.M. Roebroek


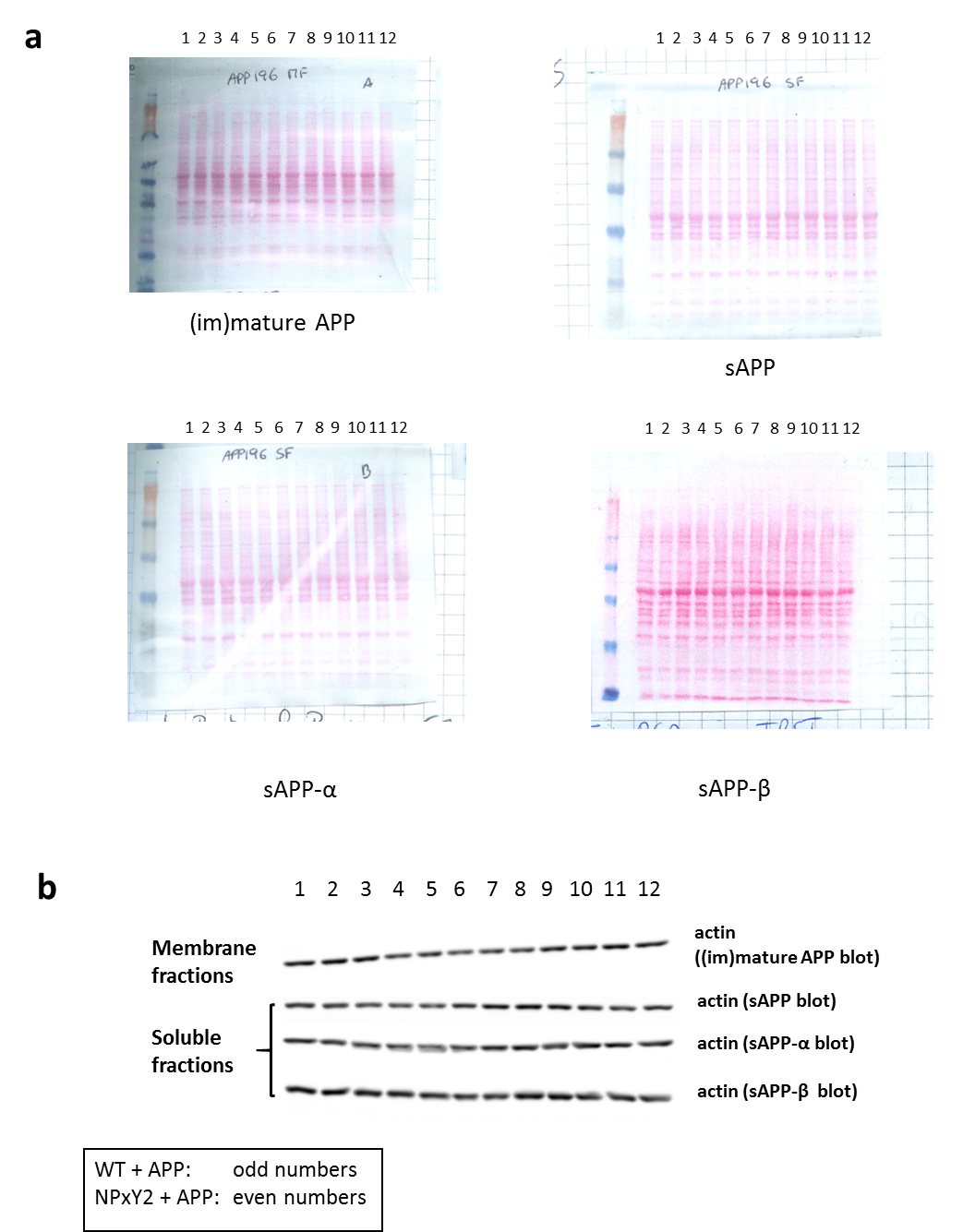


**Suppl. Fig 1. Confirmation of the analysis of equal amounts of protein in western blot analysis of the protein fractions used in the analyses showed in Fig. 3 of the manuscript. (a)** Ponceau S staining confirming the loading and transfer of equal amounts of protein to the blots used for the analysis of the expression of (im)mature APP (membrane fractions), sAPP, sAPP-α and sAPP-β (3 x soluble fractions). **(b)** Analysis of actin expression of the four blots presented in (a), reanalyzed using an antibody against actin. The blots show from left to right alternating WT + APP and NPxY2 + APP samples (in total 12 samples).

**
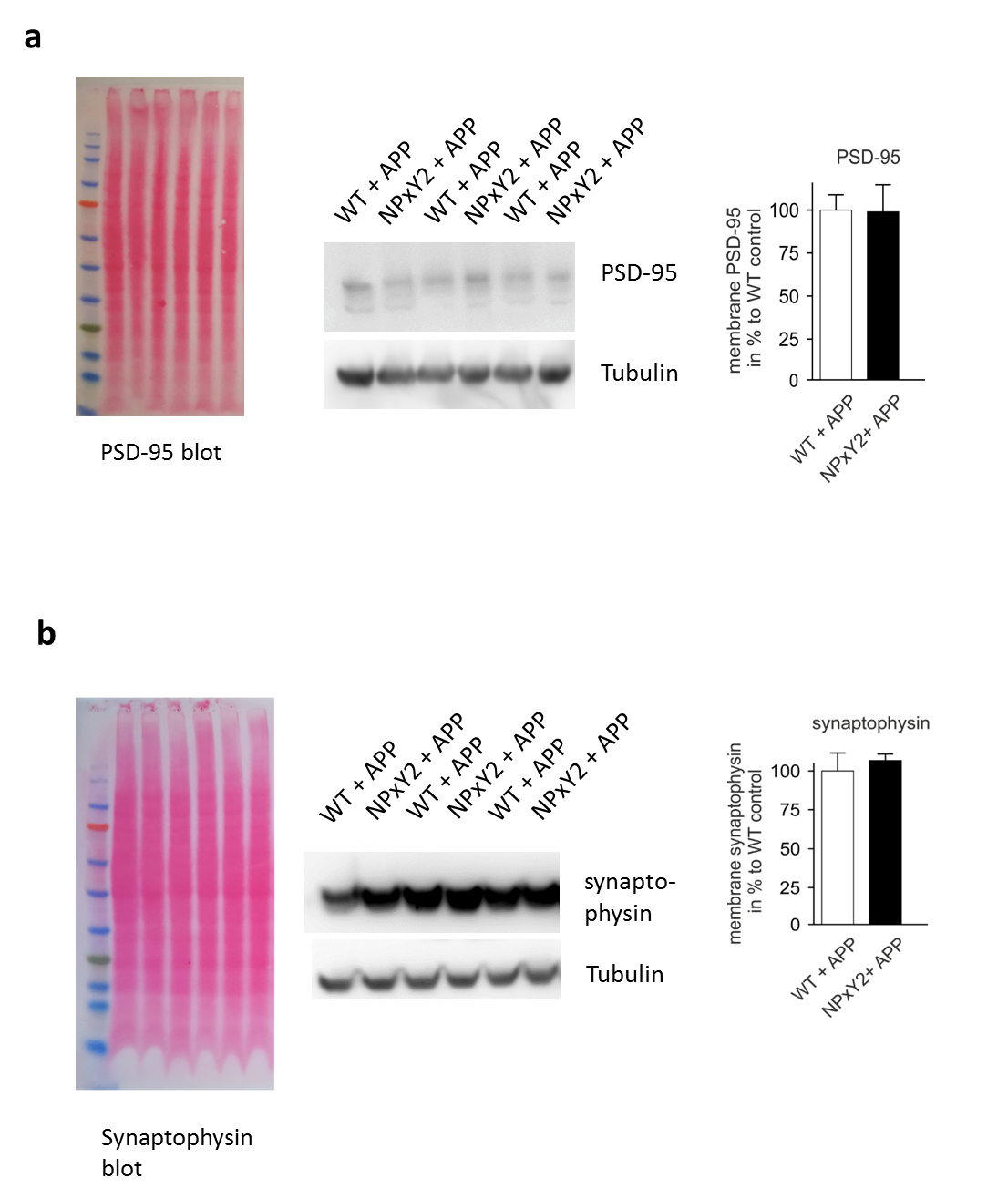
**

**Suppl. Fig. 2. Equal expression levels of the membrane proteins PSD-95 and synaptophysin as an internal control for equal loading of protein of membrane fractions. (a + b)** Ponceau S staining confirming the loading and transfer of equal amounts of protein of membrane fractions (a +b, left) to blots used for the analysis of the expression of PSD-95 (a, middle and right) and synaptophysin (b, middle and right) showing comparable expression levels of the two proteins as internal control. Additionally, it is shown, that the expression levels for tubulin are comparable. Both blots show from left to right alternating WT + APP and NPxY2 + APP samples (6 samples each blot).

**
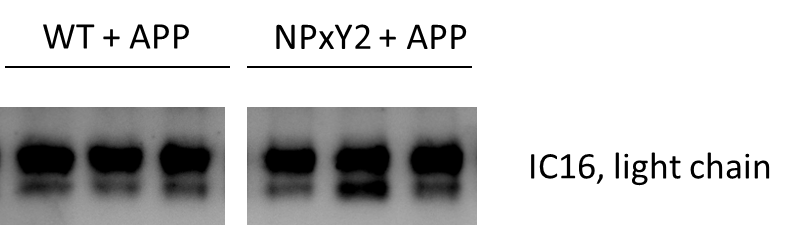
**

**Suppl. Fig. 3. Immunoblot data from the light chain of the antibody (IC 16) that was used for immunoprecipitation showing that equal amounts of antibody were used for the pulldown of Aβ.** Three representative samples for both WT + APP and NPxY2 + APP are shown, illustrating the limited variation.
